# Seeing More by Showing Less: Orientation-Dependent Transparency Rendering for Fiber Tractography Visualization

**DOI:** 10.1371/journal.pone.0139434

**Published:** 2015-10-07

**Authors:** Chantal M. W. Tax, Maxime Chamberland, Marijn van Stralen, Max A. Viergever, Kevin Whittingstall, David Fortin, Maxime Descoteaux, Alexander Leemans

**Affiliations:** 1 Image Sciences Institute, University Medical Center Utrecht, Utrecht, The Netherlands; 2 Centre de Recherche CHUS, Sherbrooke, Canada; 3 Sherbrooke Connectivity Imaging Lab (SCIL), Computer Science Department, Faculty of Science, University of Sherbrooke, Sherbrooke, Canada; 4 Department of Nuclear Medicine and Radiobiology, Faculty of Medicine and Health Science, University of Sherbrooke, Sherbrooke, Canada; 5 Department of Diagnostic Radiology, Faculty of Medicine and Health Science, University of Sherbrooke, Sherbrooke, Canada; 6 Division of Neurosurgery and Neuro-Oncology, Faculty of Medicine and Health Science, University of Sherbrooke, Sherbrooke, Canada; University Health Network and University of Toronto, CANADA

## Abstract

Fiber tractography plays an important role in exploring the architectural organization of fiber trajectories, both in fundamental neuroscience and in clinical applications. With the advent of diffusion MRI (dMRI) approaches that can also model “crossing fibers”, the complexity of the fiber network as reconstructed with tractography has increased tremendously. Many pathways interdigitate and overlap, which hampers an unequivocal 3D visualization of the network and impedes an efficient study of its organization. We propose a novel fiber tractography visualization approach that interactively and selectively adapts the transparency rendering of fiber trajectories as a function of their orientation to enhance the visibility of the spatial context. More specifically, pathways that are oriented (locally or globally) along a user-specified opacity axis can be made more transparent or opaque. This substantially improves the 3D visualization of the fiber network and the exploration of tissue configurations that would otherwise be largely covered by other pathways. We present examples of fiber bundle extraction and neurosurgical planning cases where the added benefit of our new visualization scheme is demonstrated over conventional fiber visualization approaches.

## Introduction

Diffusion magnetic resonance imaging (dMRI) is a unique technique that can infer information about the architectural organization of tissue in vivo [1,2]. It works by sensitizing the MRI sequence to the random motion of water molecules in a particular direction. Each variation in measurement direction, degree of sensitivity to diffusion, and diffusion time yields an individual image that contains unique information. These images can be combined to obtain a more complete picture of the diffusion properties, generally resulting in a complex high-dimensional dataset.

**Fig 1:**
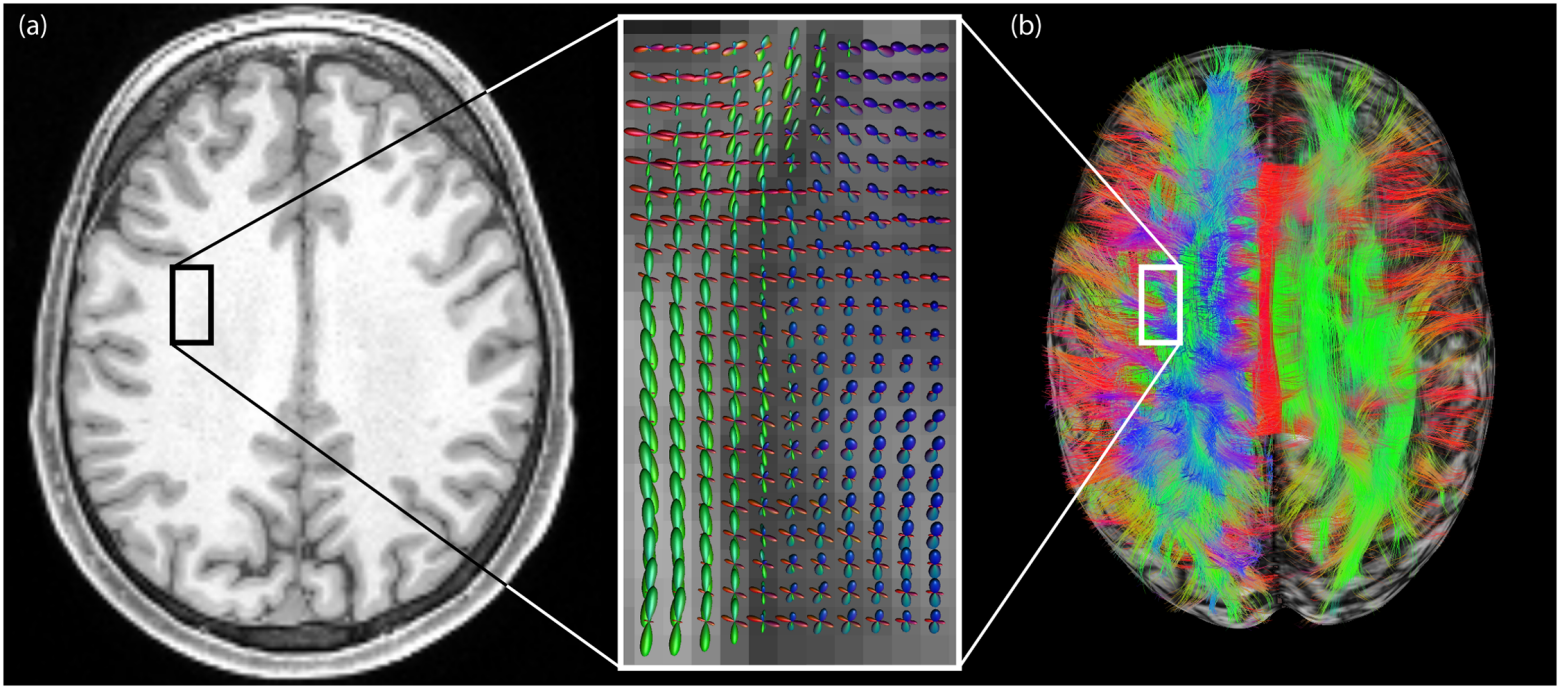
Cluttered fiber tractography visualizations due to the ability to resolve “crossing fibers” at a voxel level and the great overlap of pathways. (a) At the location of the square in the anatomical T1-weighted image (left), the dODFs (visualized as spherical “glyph” representations overlaid on a fractional anisotropy map in the magnified box on the right) reveal fiber crossings at the voxel level [42]. (b) Left hemisphere: pathways greatly overlap, resulting in a cluttered view (superior) in which underlying pathway configurations are hidden. The square indicates the same location as in (a). Right hemisphere: orientation-dependent transparency rendering in which all streamline segments that run in the direction of the viewing axis are rendered transparent. In this way, the underlying structures are revealed and can be explored.

The high dimensionality of dMRI data challenges not only image interpretation, but also its visualization [3,4]. Therefore, dMRI reconstruction methods generally attempt to reduce the amount of data to meaningful features that can subsequently be visualized. One example of such a feature is the fiber orientation distribution function (fODF), which gives the probability of a fiber population in each direction. fODFs can be visualized at every location as spherical “glyph” representations that have a magnitude proportional to the probability in every direction (Fig 1a, right) [5]. Whereas these glyph representations only contain local information, fiber tractography provides a way to visualize large-scale structures by virtually reconstructing trajectories, thereby further simplifying the data on a more global scale. Tractography visualizations are increasingly used in clinical applications, for example in neurosurgical procedures [6,7] and in brain connectivity studies. However, in contrast to conventional 2-dimensional (2D) slice-visualizations that are often used in clinical practice, tractography visualizations provide 3D information that might be hard to interpret due to streamlines that are running in the direction of the viewing axis or “out of the plane” (e.g. the geometry of the corticospinal tract (CST), which runs in inferior-superior orientation, is hard to interpret from a 2D axial slice-visualization). Therefore, several methods have been presented that focus on the 3D visualization of reconstructed fiber pathways; these are based on geometric principles like streamlines [8], streamtubes [9], hyperstreamlines [10–12], tuboids [13], triangle strips [14], and streamribbons [15]. In addition to these different geometric principles, the visualization methods also apply shadowing [16], lighting [17], and coloring.

Notwithstanding all the efforts to improve tractography visualization, displaying large tractography datasets remains highly challenging [3,18]. Such visualizations are often cluttered due to overlapping pathways in 3D views and the ability to resolve “crossing fibers” at a voxel level (Fig 1a and 1b, left) [19,20]. Therefore, it is desirable to even further focus on the relevant information available in tractography datasets. One approach is to strategically place seed regions to only extract bundles of interest [21]. While these visualizations provide greater detail, they often lose important contextual information. Other approaches have been developed that try to address the challenge of visualizing both detail and context of streamlines [3]. Schurade et al. (2010) [22] proposed a cutting surface to select pathways of interest based on their interaction with this surface, somewhat similar to real anatomical dissections. Some methods use light exchange between streamlines to show more detail in a contextual visualization [16,23]. Calamante et al. (2012) [24] proposed a technique to generate 2D “super resolved” track density maps based on the intersection between dense tractography streamlines and a user-defined sub-milimeter grid.

In this work, we propose a new visualization approach that interactively and selectively visualizes pathways based on their orientation by applying orientation-dependent transparency rendering [25]. This approach renders pathways more opaque or transparent if they run parallel to a predefined opacity axis (Fig 1b, right). In this way, a more sur veyable picture of the 3D architectural organization of these trajectories can be obtained. The proposed approach allows us to better explore the underlying tissue configurations that would be covered when using conventional visualization approaches. We explore different aspects of the method, such as local vs. global transparency rendering and choice of opacity function, and show example applications. A real-time software tool is made available that allows interactive orientation-dependent transparency rendering in large tractography datasets.

## Materials and Methods

### Orientation-dependent transparency rendering

In this work, each streamline was rendered with an opacity related to its orientation (either on a local or global scale) with respect to a predefined *opacity axis*. We will first describe our definitions of local and global streamline orientation, and subsequently propose useful opacity functions for transparency rendering in different scenarios.

#### Local vs. global streamline orientation

A streamline is a curve γ:ℝ→ℝ^3^, *t* ↦ (*x*(*t*),*y*(*t*),z(t)) with *x*(*t*), *y*(*t*), and *z*(*t*) its components at position ***r***, and denoted as *γ*(*t*) = **r**(*t*). Tractography methods yield a finite sequence of points (*γ*_*i*_)_i∈{1,2,…,N}_ to represent a streamline [8,26], where short notation *γ*(*t*_*i*_) = *γ*_*i*_ = (*x*_*i*_,*y*_*i*_,*z*_*i*_) is used. From this sequence of points we can compute the local and global orientations of a streamline.

**Fig 2:**
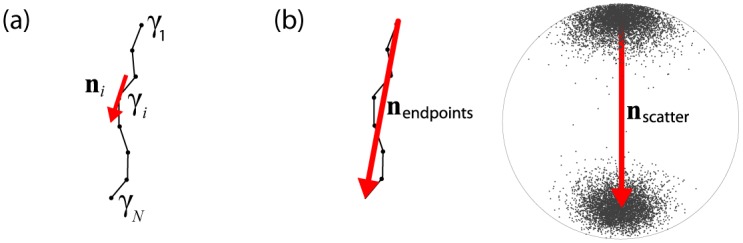
Definitions of local (a) and global (b) streamline orientation. (a) Locally, streamline orientation ***n*** *_i_* at a point *γ*_*i*_ was approximated by the normalized vector connecting its neighbors. (b) Globally, streamline orientation was approximated by 1) calculating the normalized vector that connects the endpoints of a streamline (***n***_endpoints_, left), and 2) calculating the first eigenvector ***s***_1_ of the scatter matrix ***S***, which is the maximum likelihood estimate of the mean axis of a 3-dimensional bipolar Watson distribution on the unit sphere (***n***_scatter_, right).

*Locally*, streamline orientation ***n*** *_i_* at a point *γ*_*i*_ was approximated by the normalized vector connecting its neighbors (Fig 2a):

$$n_{i}=\{\begin{array}{cc} \frac{\gamma_{i+1}-\gamma_{i-1}}{∥\gamma_{i+1}-\gamma_{i-1}∥}, & i\in \left\{2,\ldots ,N-1\right\}\\ \frac{\gamma_{i+1}-\gamma_{i}}{∥\gamma_{i+1}-\gamma_{i}∥} & i=1\\ \frac{\gamma_{i}-\gamma_{i-1}}{∥\gamma_{i}-\gamma_{i-1}∥} & i=N. \end{array}$$ (1)

*Globally*, streamline orientation was approximated in two different ways: 1) by calculating the normalized vector that connects the endpoints of a streamline (Fig 2b, left):

$$n_{\text{endpoints}}=\frac{\gamma_{N}-\gamma_{1}}{||\gamma_{N}-\gamma_{1}||}\;,$$ (2)

and 2) by calculating the scatter matrix ***S***, which is the average of the dyadics of all local orientations ***n*** *_i_* within a streamline:

$$S=\frac{1}{N}\sum_{i=1}^{N}n_{i}n_{i}^{T}.$$ (3)

In the latter case, the first eigenvector of ***S***, i.e., ***s***_1_, approximates the global streamline orientation:

$$n_{\text{scatter}}=s_{1}.$$ (4)

This is similar to considering the vectors ***n***_*i*_ as samples of a 3-dimensional bipolar Watson distribution on the unit sphere *S* ^2^ (Fig 2b right), with *S* = {***n***|***n***∈ ℝ^3^,||***n***|| = 1} (Appendix A) [27]. The maximum likelihood estimate of the mean axis ±$\hat{\mu }$ of the Watson distribution is the first eigenvector ***s***_1_ of ***S***

In addition, the eigenvalues *β*_1_, *β*_2_, *β*_3_ of ***S*** can provide useful information on the shape of the data [27]. When *β*_1_ is significantly larger than *β*_2_ and *β*_3_, the data is well described by a bipolar/bimodal distribution, and we can consider ***s***_1_ to be a reasonable estimate of the streamline orientation. To investigate whether this assumption holds, one could look at the linear coefficient

$$c_{l}=\frac{\beta_{1}-\beta_{2}}{\beta_{1}+\beta_{2}+\beta_{3}},$$ (5)

which ranges from 0 to 1, and is close to 1 in the case of *β*_1_ ≫ *β*_2_ ≅ *β*_3_ [28]. The *c*_*l*_ measure can be computed for each streamline, and can be used as an extra tuning parameter for global transparency rendering (see Section “Streamline dispersion in global transparency rendering”).

#### Opacity functions

**Fig 3:**
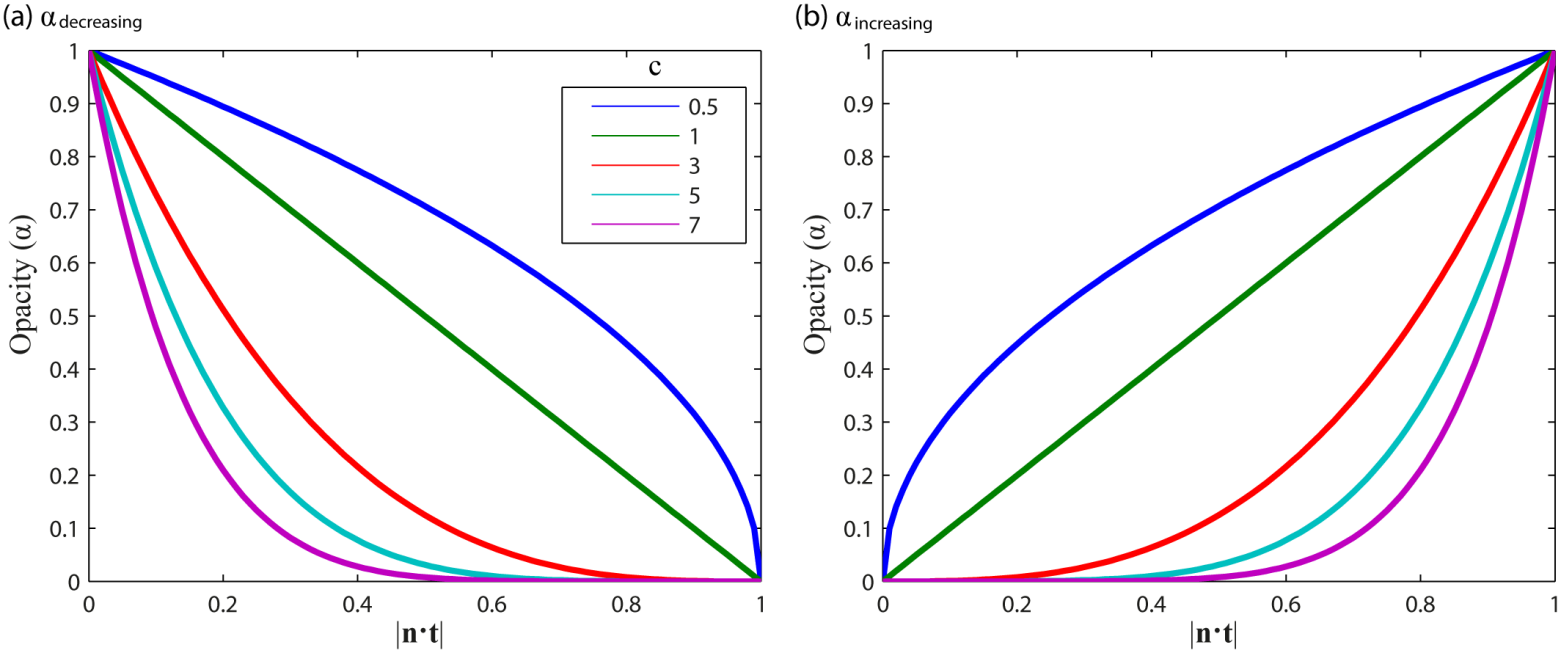
Two opacity functions and the influence of tuning constant ***c***. (a) *α*_decreasing_: the opacity is decreasing when the streamline orientation is increasingly parallel to the opacity axis (i.e. when the inner product |***n*·*t***| is increasing, or the angle *θ* between ***n*** and ***t*** is decreasing), and (b) *α*_increasing_: the opacity is increasing when the streamline orientation is increasingly parallel to the opacity axis.

The opacity (*α*) at a point *γ*_*i*_ lies in the interval [0, 1], where an *α* value of 1 signifies fully opaque, and a value of 0 signifies fully transparent. In this study, the opacity is considered a function of the streamline orientation ***n*** (with ***n*** = ***n***_*i*_ in the local and ***n*** = ***n***_*endpoints*_ or ***n*** = ***n***_*scatter*_ in the global case) and an *opacity axis* ***t***. We propose two kinds of opacity function (Fig 3): 1) the opacity is decreasing when the streamline orientation is increasingly parallel to the opacity axis (i.e. when the inner product |***n***·***t***| increases, or the angle *θ* between ***n*** and ***t*** decreases, Fig 3a):

$$\alpha_{\text{decreasing}}\left(\gamma_{i}\right)={\left(1-\left|n\cdot t\right|\right)}^{c};$$ (6)

or 2) the opacity is increasing when the streamline orientation is increasingly parallel to the opacity axis (Fig 3b):

$$\alpha_{\text{increasing}}\left(\gamma_{i}\right)={\left|n\cdot t\right|}^{c}.$$ (7)

Here, *c* is a constant that tunes the “steepness” of the curve and the “cut-off” value of |***n*·*t***| where the opacity starts to approach 0.

To motivate our choices of opacity function, we distinguish two scenarios: 1) the opacity axis coincides with the *viewing axis* ***v***, which is the axis normal to the screen (***t***||***v***), and 2) the opacity axis does not coincide with the viewing axis (***t***∦***v***). In the first case, streamlines that run parallel to the viewing axis do not provide any additional information to the user but obscure underlying streamlines instead. Therefore, it is useful to render such streamlines less opaque (using *α*_decreasing_) since the 2D projection of such a line results in a single point. In the second case, it is useful to visualize streamlines that run in a particular direction and render the other ones transparent (using either *α*_decreasing_ or *α*_increasing_). In this case, the opacity axis is fixed in a predefined direction and the rendered streamlines can be viewed from different angles.

#### Streamline dispersion in global transparency rendering

In the global case, the *c*_*l*_ measure indicates how “dispersed” the pathway is (i.e., a low value of *c*_*l*_ indicates that the streamline does not run in one dominant direction). When a pathway travels in many different directions, rendering the streamline opaque might be desired to be able to see it in all views. Therefore, we investigated the influence of a “*c*_*l*_ threshold” $T_{c_{l}}$ on the opacity function: If the *c*_*l*_ of a pathway is below the threshold, render the current pathway opaque (i.e., *α* = 1); otherwise, apply the opacity function *α*_decreasing_ or *α*_increasing_.

#### Real-time implementation

An interactive implementation of the orientation-dependent transparency rendering method is made available in the Fibernavigator (www.github.com/chamberm/fibernavigator) [21]. This software allows real-time visualization of whole brain fiber tractography datasets, as well as instantaneous segmentation of user-defined bundles. The computation is done in C++ while the rendering is done with calls to OpenGL and GLSL shaders.

For real-time orientation-dependent transparency rendering, the line segments of the streamlines are first ordered according to their mean distance from the point of observation to prevent rendering artifacts. As the viewing axis changes, this ordering is recalculated on the fly, which makes the efficiency of the method proportional to the number of line segments that needs be sorted. To accelerate this depth-sorting step, tractography datasets were compressed by removing redundant points in regions where a streamline is almost linear (tolerance error of 0.01 mm was used) [18]. Finally, an opacity value is attributed to each point according to Eq (6) or Eq (7).

### Data acquisition and processing

To illustrate our method, two tractography datasets were used. The first dataset was a Human Connectome Project (HCP) dMRI dataset with an isotropic voxel size of 1.25 mm [29,30]. Only the b = 3000 s/mm^2^ shell with 90 diffusion directions was used. Whole brain deterministic tractography was performed with *ExploreDTI* [31,32] using constrained spherical deconvolution (CSD, lmax = 8 [33]) and recursive calibration of the response function [34]. The second dataset was from a tumor patient (42 year old, male) with an oligoastrocytoma anaplastic tumor (Grade III, WHO) located in the left prefrontal cortex. The study was approved by the Internal Review Board of the Centre Hospitalier Universitaire de Sherbrooke (CHUS) and performed according to their guidelines. Written informed consent was obtained from the patient for the use of anonymized data. Acquisition parameters were: voxel size 2 x 2 x 2 mm^3^, b = 1000 s/mm^2^, 64 directions (further details have been previously described in Bernier et al., 2014 [35]).

## Results

This section is organized according to the scenarios we have considered previously: either the opacity axis coincides with the viewing axis, or the opacity axis does not coincide with the viewing axis. We use these scenarios to show different aspects of orientation-dependent transparency rendering, including local vs global streamline orientation, choice of opacity function, and the role of streamline dispersion. Table 1 shows an overview of the different options, of which we will show some in this section (references to the corresponding figures can be found in Table 1). The possibility to interactively rotate the opacity and viewing axes and switch between different settings can be appreciated in the supplementary video available online (https://www.youtube.com/watch?v=IzJ537KNpR0).

**Table 1:** Overview of the different options in orientation-dependent transparency rendering.

		Opacity function	Relation of viewing and opacity axis	
			*t*||*v*	*t*∦*v*
**Scale**	* Local*	*α*_decreasing_	Fig 4b	
		*α*_increasing_		
	* Global*	*α*_decreasing_	Fig 5	Fig 6a
		*α*_increasing_		Fig 6b

We investigated different scales (either local or global transparency rendering), different opacity functions (either decreasing or increasing with increasing inner product of streamline orientation and opacity axis |***n***·***t***|), and different relationships between opacity axis ***t*** and viewing axis ***v*** (either ***t***||***v*** or ***t***∦***v***). We show some of these combinations in the referred figures.

### Opacity axis coincides with viewing axis

#### Local streamline orientation

**Fig 4:**
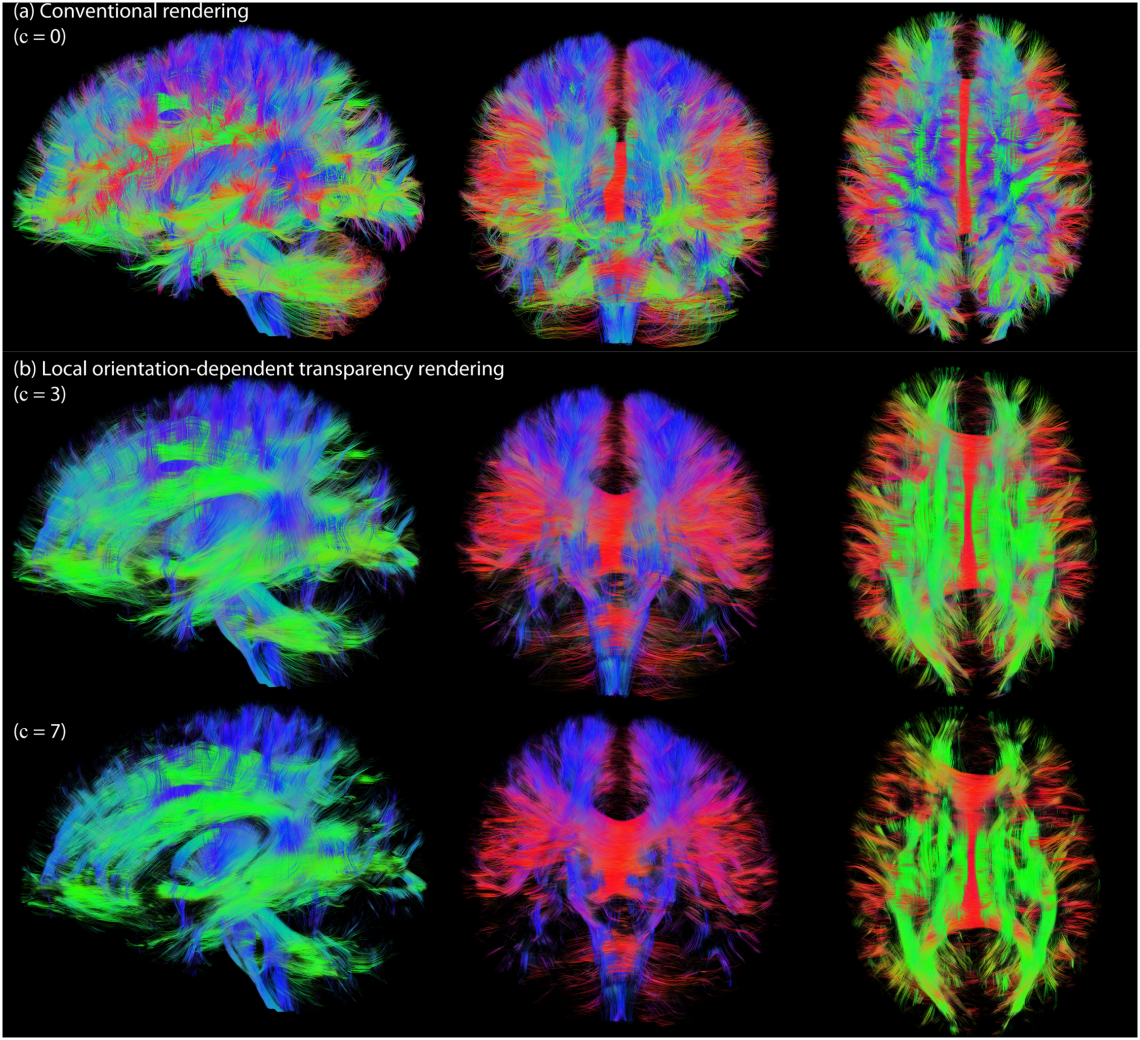
Comparison of conventional streamline rendering (a) with local orientation-dependent transparency rendering (b). Here, the opacity axis coincides with the viewing axis and *α*_decreasing_ is applied. The middle and bottom rows show results for different tuning constants (*c* = 3 and *c* = 7).

Fig 4 compares conventional streamline rendering (a) with local orientation-dependent transparency rendering (b) where the opacity axis coincides with the viewing axis (*α*_decreasing_, Eq (6)). Pathway segments that are more aligned with the viewing axis are rendered more transparent. This greatly improves the visualization of the underlying tissue configuration oriented perpendicular to the viewing axis. The middle and bottom rows show results for different tuning constants (*c* = 3 and *c* = 7). When increasing the tuning constant from *c* = 3 to *c* = 7, more streamline segments are rendered transparent due to the increased “steepness” and lower |*n*·*t*| “cut-off” value of the opacity function (*α*_*decreasing*_, see also Fig 3(a)).

#### Global streamline orientation

**Fig 5:**
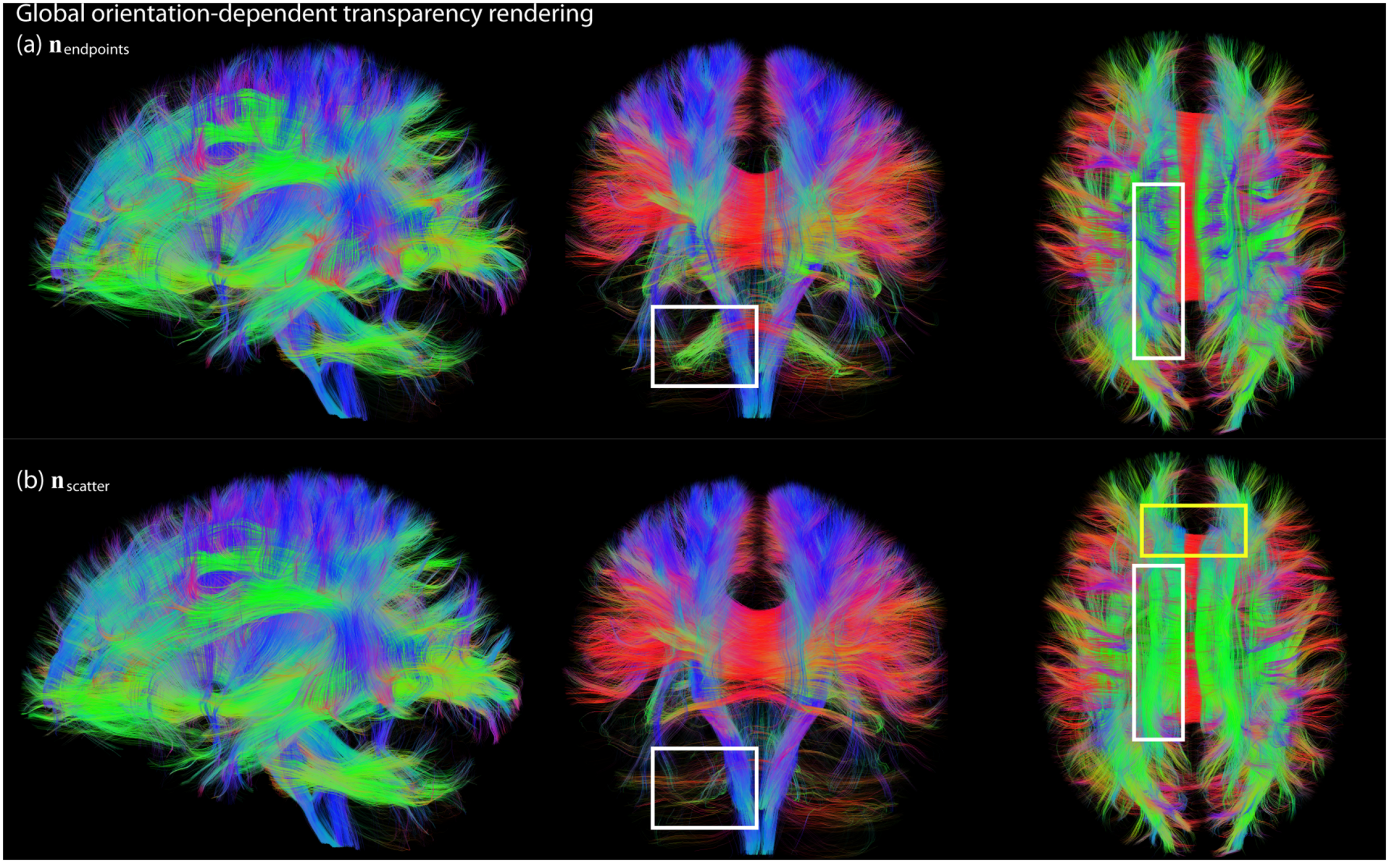
Global transparent streamline rendering (*c* = 3, *α*_decreasing_), in which the opacity is the same along the whole streamline (yellow square shows that the whole Cg bundle is visible). In (a) the mean direction of each streamline is based on the endpoints (***n***_endpoints_, Eq (2)), whereas in (b) the mean direction is calculated from the scatter matrix (***n***_scatter_, Eq (4)). The white squares indicate differences between both methods.

The results of global transparent streamline rendering are shown in Fig 5. Whereas for local transparency rendering the opacity is different for every line segment (Fig 4b), for global transparency rendering the opacity is the same along the whole streamline. The latter better preserves the continuous character of a streamline. For example, the whole cingulum (Cg) bundle is visible with global transparency rendering (Fig 5b, yellow square in axial view), whereas parts that run in inferior-superior direction are rendered transparent with local transparency rendering (Fig 4b, same location). Fig 5a and 5b compare the two different methods to obtain the global streamline orientation, based on the endpoints (***n***_endpoints_, Eq (2)) and based on the scatter matrix (***n***_scatter_, Eq (4)), respectively. The main difference between the methods can be observed in the transparent rendering of U-shaped streamlines (e.g. in the corpus callosum (CC) and in the middle cerebellar peduncle, indicated with white squares in axial views and coronal views, respectively). In the CC, for example, the global orientation of the streamlines is left-right when only looking at the endpoints, whereas it is inferior-superior when looking at the first eigenvector of the scatter matrix (i.e., more line segments are oriented along inferior-superior direction). This explains why the U-shaped CC streamlines are still visible in Fig 5a (blue streamline segments in the white squares), whereas they are rendered transparent in Fig 5b (and thus do not “obscure” other bundles). These scenarios already indicate that there might not be a well-defined global direction for such fibers, which we will investigate further in Section “Streamline dispersion in global transparency rendering”.

### Opacity axis does not coincide with viewing axis

#### Opacity functions

**Fig 6:**
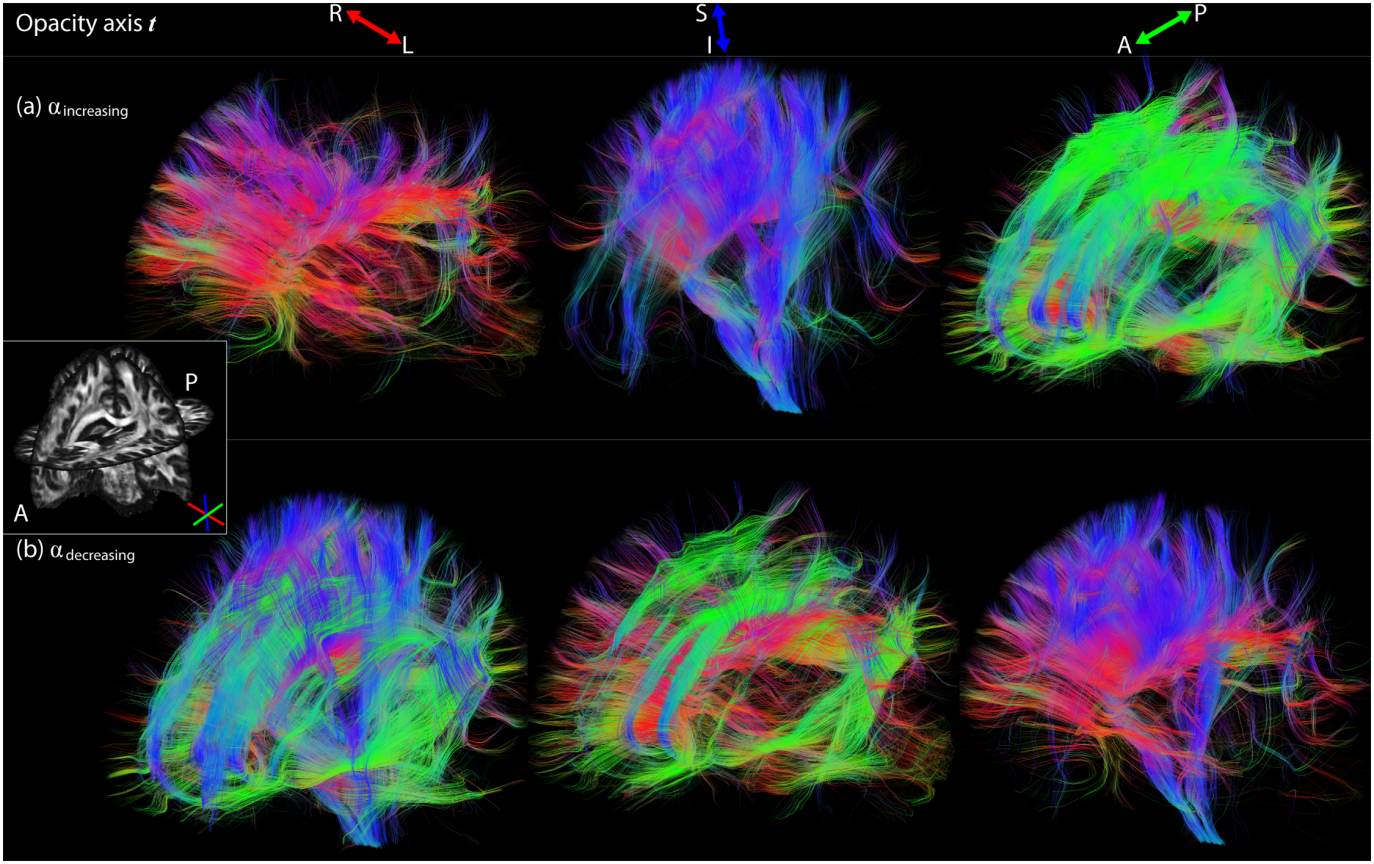
Opacity axis is not aligned with the viewing axis, but fixed along the left-right (left), inferior-superior (middle), or antero-posterior (right) axis. Frames (a) and (b) illustrate the difference between the two opacity functions *α*_decreasing_ (Eq (6)) and *α*_increasing_ (Eq (7)) on global transparency rendering (***n***_scatter_, *c* = 10), respectively. Streamlines can either be rendered opaque (a) or transparent (b) along a specific axis.

Fig 6 shows an oblique view in which the opacity axis does not coincide with the viewing axis but is defined along the left-right (left), inferior-superior (middle), and antero-posterior (right) axes. Fig 6a and 6b illustrate the difference between the two opacity functions *α*_increasing_ (Eq (6)) and *α*_decreasing_ (Eq (7)) on global transparency rendering, respectively. When applying *α*_increasing_, streamlines that increasingly coincide with the opacity axis are rendered more opaque. This can be used to only *show* pathways that run in a particular direction (Fig 6a). On the other hand, when applying *α*_decreasing_, streamlines that increasingly coincide with the opacity axis are rendered more transparent. This can be used to only *eliminate* pathways that run in a particular direction (Fig 6b).

#### Streamline dispersion in global transparency rendering

**Fig 7:**
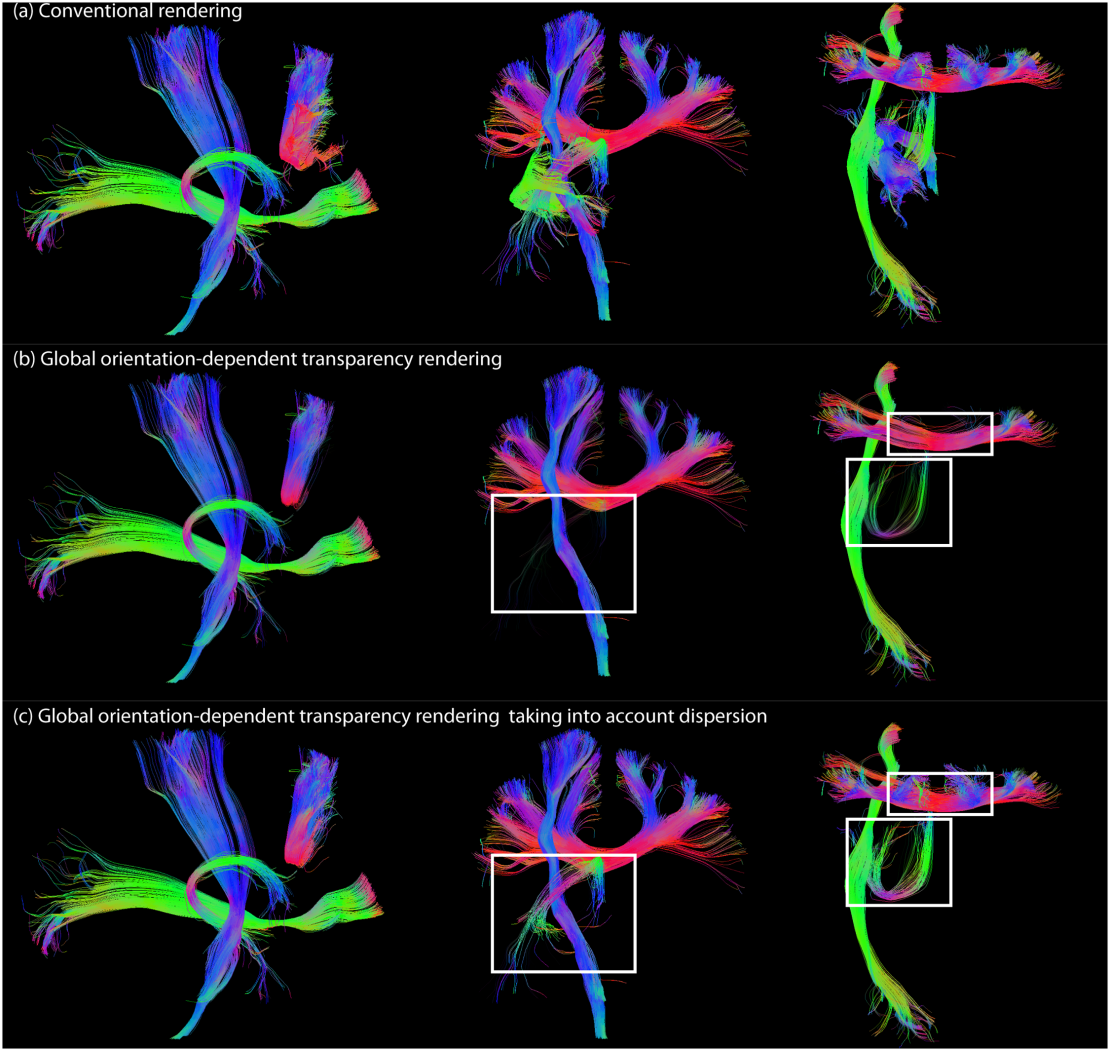
The effect of taking into account streamline dispersion on a set of extracted bundles: left iFOF, left CST, CC, and left fornix. (a) Conventional rendering in a sagittal, coronal, and axial view, respectively. (b) Global orientation-dependent transparency rendering (opacity axis coincides with the viewing axis with *α*_*decreasing*_, *c* = 3, and *n*_*scatter*_). (c) Global transparency rendering (same settings as in (b)) taking into account the dispersion of the streamlines ($T_{c_{l}}=0.29$). White squares highlight streamlines that are rendered transparent when not taking into account streamline dispersion, but are visible when applying a *c*_*l*_ threshold.

Strongly dispersed streamlines do not have a single and well-defined global direction, and it might therefore be beneficial to render them opaque to be able to see them in all views (see also Fig 5). Fig 7 shows the results for taking into account streamline dispersion on a set of (both curved and straight) streamlines of the left inferior fronto-occipital fasciculus (iFOF), left corticospinal tract (CST), CC, and left fornix. Fig 7a shows conventional rendering (no transparency) of these bundles in a sagittal, coronal, and axial view, respectively. Fig 7b shows global orientation-dependent transparency rendering. Straight streamlines that have a global orientation along the viewing axis are rendered transparent (lateral projections of the CC, IFOF, and CST in respectively the sagittal, coronal, and axial view), but also curved streamlines of the fornix are rendered (almost) transparent in both views, and the U-shaped streamlines of the CC are rendered transparent in the axial view. Fig 7c shows global transparency rendering, but now we do take into account the dispersion of the streamlines. With this option, the curved fibers (fornix and U-fibers of the CC) remain visible in each view.

S1 Fig illustrates the influence of $T_{c_{l}}$ on the visualization of streamlines in the CC and the Cg. Streamlines of the CC and the Cg are relatively curved, so the orientational distribution of their line segments will be more “dispersed”. The parameter $T_{c_{l}}$ can for example determine to what extent the fanning of the CC is visualized (S1a Fig).

### Applications

In the previous sections, we have shown that orientation-dependent transparency rendering greatly contributes to exploring underlying tissue configurations that would otherwise be covered by other pathways. In this section, we present two applications in which orientation-dependent transparency rendering is useful: the extraction of fiber bundles, and the combined visualization of streamlines with space occupying regions such as tumors.

#### Bundle localization and extraction

**Fig 8:**
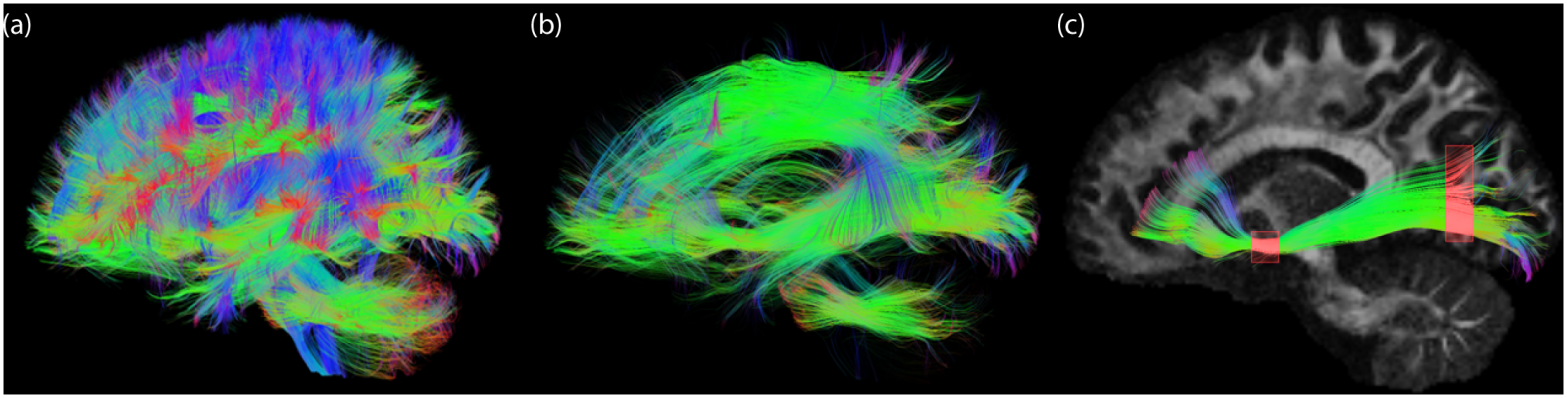
Bundle extraction with orientation-dependent transparency rendering. (a) Conventional whole brain fiber tractography rendering. Using orientation-dependent transparency rendering, streamlines that are running parallel to the antero-posterior axis can easily be visualized (b, *α*_increasing_ with *c* = 7). From this visualization, one can easily extract the iFOF by positioning two ROIs (red boxes, stem and V1 area).

Fig 8a shows a conventional whole brain fiber tractography rendering, in which the more superficial streamlines obscure the deeply located streamlines. In this view, it is for example difficult to accurately localize the inferior fronto-occipital fasciculus (iFOF). When using orientation-dependent transparency rendering, only streamlines running parallel to the opacity axis (in this case antero-posteriorly) will be maintained, and as a result, the iFOF is clearly identifiable (Fig 8b). By interactively positioning regions of interest (ROIs) at the stem [36] of the iFOF and in V1 (Fig 8c), the bundle of interest can be extracted.

#### Visualization of space occupying regions: Neurosurgical application

**Fig 9:**
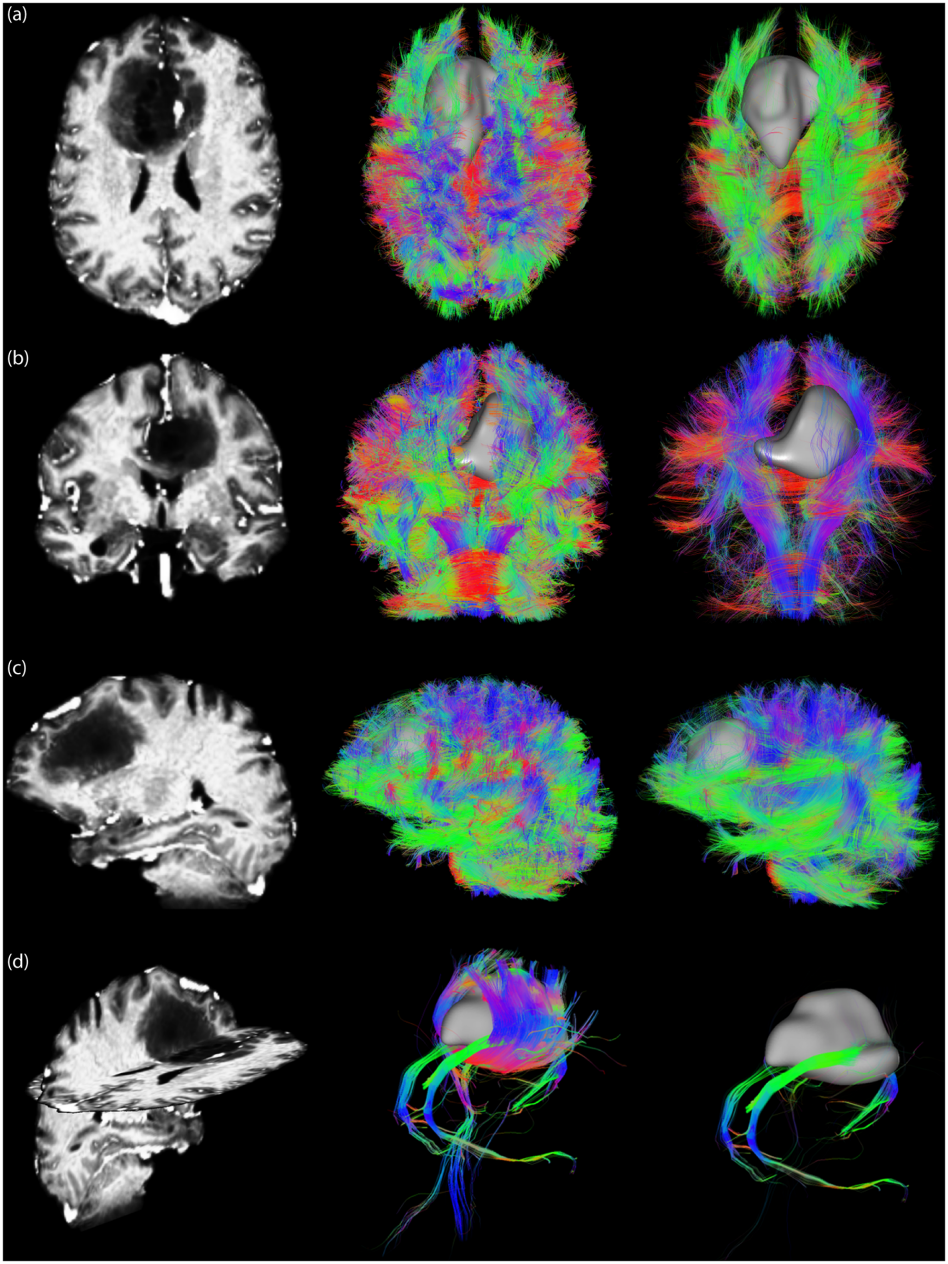
Neurosurgical application of the orientation-dependent transparency rendering method. The left column shows a high resolution T1-weighted image for anatomical reference. The middle column shows conventional streamline rendering, where the tumor (gray) is largely covered by the massive amount of streamlines. The right column shows that orientation-dependent transparency rendering (*α*_decreasing_ with *c* = 4) allows for in-depth visualization of a tumor mass by rendering streamlines that are running towards the viewing axis transparent. Fig (a), (b) and (c) show superior, anterior, and lateral views, respectively. In (d), the tumor volume is used as an ROI for the selection of relevant streamlines. By fixing the opacity axis along the antero-posterior direction (*α*_increasing_ with *c* = 4), one can render Cg streamlines opaque, showing that they penetrate the tumor area.

Fig 9 shows a neurosurgical application of the orientation-dependent transparency rendering method. The left column shows the tumor location on an anatomical T1-weighted image, the middle column shows conventional streamline rendering, and the right column shows orientation-dependent transparency rendering. The superior (a), anterior (b) and lateral (c) views show that in conventional rendering, the tumor is largely covered by the massive amount of streamlines that are running parallel to the viewing axis. In contrast, orientation-dependent transparency rendering enables clearer visualization of the tumor mass by rendering streamlines that are running towards the viewing axis more transparent. Fig 9d shows only the streamlines that touch the segmented tumor volume. By fixing the opacity axis along the antero-posterior direction, one can only render Cg streamlines opaque. The resulting view shows that the Cg pathway infiltrates the tumor area, which would otherwise have been covered by the superior projections of the CC.

## Discussion

We propose a new visualization approach to interactively and selectively visualize pathways based on their orientation, resulting in a less cluttered and obscure picture of the 3D architectural organization of streamline trajectories. While other software packages have the possibility to attribute coloring and/or opacity levels to streamlines (e.g., [31,37,38]), we have explored different aspects of real-time orientation-dependent transparency rendering such as local vs global transparency, choice of opacity function, and the role of streamline-dispersion. In addition, we have shown two example applications. Below, we will discuss the limitations, potential improvements, and future perspectives of the method.

### Methodology

#### Local vs. global streamline orientation

We have shown results for both local and global transparency rendering (Figs 3b and 4, respectively). The advantage of global rendering is that the opacity is the same along the pathway, which better preserves its continuous character. The drawback is that globally opaque streamlines can still largely obscure underlying pathways. In addition, it can be challenging to define a robust and representative global orientation of a streamline, especially in the case of highly curved and dispersed fibers. We have investigated two approaches to define global pathway orientation (Fig 5): either based on the endpoints, or based on the scatter matrix of the line segments of a pathway. The endpoints-approach gives useful information as to what parts of the brain are connected by a particular pathway, and is often used in dMRI (visualization) packages to define the global color of a pathway (e.g. [21,39]). However, the location of the endpoint can be heavily influenced by accumulated errors in streamline propagation (e.g. due to noise), in which case it might be beneficial to use the scatter-approach. The endpoints- and scatter-approaches give different results in the case of curved U fibers (see Fig 5). The scatter approach can give an indication of the dispersion/linearity of a pathway, which can be used as an extra parameter to tune opacity and to reduce the risk of hiding important neuroanatomical details (Fig 7). Here, we have used the *c*_*l*_ measure, but there are other (dispersion) measures that can be used as an indicator of the shape of the distribution (e.g., [28,40]). More general, the opacity can be a function of the opacity axis ***t*** and the eigenvectors and eigenvalues of the scatter matrix ***S***. In this way, not only the linearity of a pathway can be characterized, but for example also its planarity. By interactively switching between local and global transparency rendering, the different methods for global streamline orientation, and different values for $T_{c_{l}}$, one can adapt the visualization to the demands of a specific application.

#### Opacity functions

The opacity functions in this work are based on power functions, in which the exponent *c* (here we choose *c*>1) can be used to tune the relationship between **|*n*·*t*|** and opacity *α*. While this opacity function is intuitive and elegant as it only has a single parameter and lies between 0 and 1 for **|*n*·*t*|** ∈ [0,1], it might be too simplistic and ad hoc. For example, increasing the exponent *c* determines at the same time the “steepness” of the curve and the “cut-off” value of **|*n*·*t*|** where the opacity starts to approach 0. Other functions with more parameters can be used instead, such as sinusoidal functions or a linear function. A linear function (*a*(*x*) = *ax*+*b*) might be more useful than sinusoidal functions since it has the freedom to easily tune the steepness and cut-off value while constraining the minimum and maximum *α* to be 0 and 1, respectively. S2 Fig shows an example of local transparency rendering with *α* a linear function of the angle *θ* = acos**|*n*·*t*|**, for different values of *a* and *b*. The cut-off value of **|*n*·*t*|** appears to have the largest influence on the visualization.

Instead of using predefined opacity functions and exploring the influence of their parameters on the visualization, one could also make a more informed decision about the preferred shape of the opacity function. To this end, one could inspect histograms of **|*n*·*t*|** as a function of the opacity axis to get an impression of the distribution of **|*n*·*t*|**. S3 Fig shows such histograms for locally (a) and globally (b) defined orientations, for opacity axis in left-right (left), antero-posterior (middle), inferior-superior (right) direction. The opacity function could then be adapted interactively (e.g. by defining it as a piecewise linear function from user defined points) to emphasize or eliminate streamlines or streamline segments with particular orientations.

#### Real-time implementation

While the static images presented in this paper give a good impression of the usefulness of orientation-dependent transparency rendering, the method comes to its full justice when interactively rotating the view and exploring different settings. To this end, we have developed a real-time implementation which is made freely available in the Fibernavigator [21]. Over 3·10^6^ line segments (over 1·10^6^ compressed) could be ordered and rendered real-time with an acceptable frame rate of over 20 frames-per-second. We found that transparency renderings were the most useful when the number of overlapping line segments was not too high (i.e., the tractography dataset was not too dense), hence we subsampled the first dataset from ~ 8·10^5^ streamlines to ~ 4·10^5^ streamlines. A possible improvement to the interactive method presented here is to accelerate the line segment depth-sorting step, enabling even smoother interaction with larger tractography datasets. Additionally, tube rendering [9] can provide more perceptual depth, since lightning techniques can be more easily applied to tuboïds than to thin lines. Future developments will include an upgraded version of the opacity-rendering method which will be implemented in MI-Brain (www.imeka.ca/mi-brain), a streamline-visualization and interaction tool based on the MITK platform (www.mitk.org).

### Applications

#### Bundle localization and extraction

The extraction of streamline bundles is most commonly done by placing ROIs [31,38] on a reference image (e.g. T1, T2, direction encoded color FA, etc.) based on anatomical knowledge. This method has the disadvantage of not showing streamlines on the fly, and thus segmentation is often done in a “blind” manner. In contrast, the ability to interactively position 3D ROIs and real-time tractography allows for fast extraction of specific pathways and direct interaction with streamlines [21]. Nevertheless, it can still be a complex task to place such ROIs when the streamline bundle of interest is obscured by other bundles. With orientation-dependent transparency rendering, one can generate a more efficient view of the current dataset, which can greatly help in ROI positioning. For example, the iFOF is a bundle that predominantly runs in the antero-posterior direction, and is often hidden by more superficial streamlines (Fig 8). Using this a priori knowledge, it is possible to render streamlines that run in this particular direction more opaque. This greatly reduces the amount of streamlines displayed, while exposing large parts of the iFOF, and thus eases the ROI positioning process. In addition, orientation-dependent transparency rendering clearly reveals a recently reported new pathway in the orbitofrontal / prefrontal cortex [41] (see e.g. Fig 5, sagittal view). This pathway has previously been undocumented because of the complex “crossing fiber” architecture in this area. New visualization techniques, such as transparency rendering, along with progress in acquisition and data processing aid in localization and identification of such previously undocumented structures.

#### Visualization of space occupying regions

In some applications, tractography results are visualized together with volume renderings. These volume renderings can become largely covered by the large amount of streamlines in tractography datasets. Combining volume renderings with orientation-dependent transparency rendering of streamlines gives a less cluttered view and allows for better exploration of the data, especially in the case of volume renderings that are located deeply in the brain. Here, we discuss two potential applications in which transparency rendering might be beneficial: a neurosurgical application (see also Fig 9), and combined dMRI and functional MRI visualization.

Neurosurgical planning requires a generalized view of deviating and infiltrating streamlines in proximity of the tumor. We found that global transparency rendering was the most appropriate in this case, since the whole streamline has the same opacity (Fig 9). Local transparency rendering might lead to misinterpretation in this case (e.g. local line segments interpreted as streamlines that terminate in the white matter). Furthermore, the ability to interact in real-time with the view and settings is necessary in pre-operative planning applications. More specifically, a neurosurgeon should be able to easily switch between conventional and opacity rendering at any time, so that no relevant pathways can be missed.

Traditional ways of coupling functional MRI (fMRI) and dMRI often come down to displaying fiber pathways that interconnect distant fMRI activation regions [6,35,42,43] (i.e. brain networks). This provides a clear and simple view of the current network of interest. However, one may be interested in looking at a more extensive (whole brain) tractogram together with fMRI clusters, to see more contextual information. Therefore, it can become challenging to spot deeply-located fMRI regions (e.g. sub-cortical activations) that are surrounded by many streamlines. To overcome this problem, orientation-dependent transparency rendering can be applied. By rendering streamlines that are running parallel to the viewing axis more transparent, an uncluttered view can be achieved for the 3D exploration of such deep fMRI regions while maintaining contextual information.

### Recommendations and future perspectives

In a nutshell, orientation-dependent transparency rendering can be used to simplify the visualization of dense streamline datasets. We have proposed different opacity functions and opacity axes settings that can be combined to achieve a particular visualization. While we believe that the power of the method lies in the possibility to interact in real-time with these different views and settings, we found transparency rendering particularly useful in two scenarios. 1) The user has *a priori* knowledge on the general direction of a bundle of interest and wants to identify and extract this bundle. In this case, one can use *α*_increasing_ (Eq (7)) with ***t***∦***v*** to inspect the bundle from different viewpoints. Global transparency rendering gives the most intuitive visualization here, since the opacity is the same along the whole streamline. 2) The user wants to do a global or more local (e.g. only the centrum semiovale) exploration of the streamline network and wants to eliminate streamlines (or segments) that run in the line of view and obscure underlying configurations. In this case, one can use *α*_decreasing_ (Eq (6)) with ***t***||***v*** and local or global opacity rendering to remove clutter. In the case of combined visualization of streamlines with volume renderings (such as tumors), we found both scenario 1) and 2) useful: the user can inspect whether a particular bundle intersects with the tumor volume, and the user can remove cluttering pathways in the line of view to obtain a more clear view of the streamlines in proximity of the tumor. While this work focuses on the presentation of the method and showcasing its different aspects, future work will further assess the (clinical) usefulness of this method in the form of a survey among highly qualified clinicians. In addition, the proposed rendering method will be integrated as part of neuronavigation software used for surgical interventions where neurosurgeons explore the wiring of the brain interactively with the tip of their neurotracking tool.

**Fig 10:**
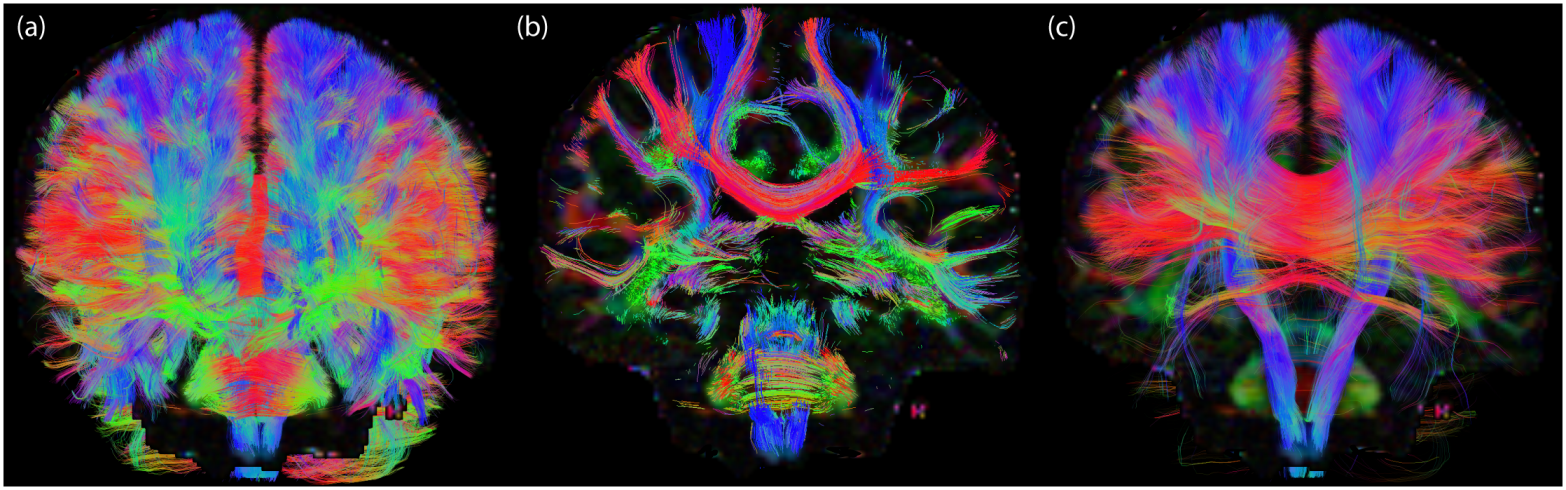
Visual comparison between three streamline rendering techniques. (a) Conventional whole brain streamline rendering in which pathways greatly overlap and clutter the view. (b) Track density imaging [24] in which only streamlines crossing a user-defined plane (in this case a coronal slice) are shown. (c) Orientation-dependent transparency rendering in which all pathways that run in the direction of the viewing axis are rendered transparent. Underlay: directionally-encoded fractional anisotropy map.

As fiber tractography yields complex 3D structural information (Fig 10a), it is challenging to combine such information with conventional 2D slice-visualizations that are currently used in clinical practice. One way is to visualize 2D track density maps (Fig 10b) [24], which show the intersection of tractography streamlines with a particular slice. While this method results in striking high-resolution images, it abandons part of the 3D depth information, and the user still has to “scroll” through the slices to get a 3D overview. Orientation-dependent transparency rendering with *α*_decreasing_ (Eq (6)) and ***t***||***v*** (Fig 10c) can in a way be interpreted as a trade-off between conventional visualization and track density slice visualization: On the one hand it shows a less cluttered view by removing pathways (or segments) that run along the viewing axis, while on the other hand it maintains information on the 3D architecture of streamlines.

## Conclusions

Orientation-dependent transparency rendering of streamlines as presented in this work avoids cluttered and obscure visualizations, and provides a way to interactively explore the 3D architectural organization of streamlines. We have explored global and local transparency rendering, different opacity functions, different settings for the opacity axis, and the role of streamline dispersion. Using orientation-dependent transparency rendering, streamlines that are oriented along the viewing axis can be rendered more transparent, thereby not obscuring underlying pathways. Alternatively, exclusively bundles that are oriented along particular axes can be visualized, virtually eliminating all other pathways. We have shown that the method is particularly useful in applications like bundle extraction and combined visualization with volume renderings such as tumors in neurosurgical planning.

## Appendix A

The Watson distribution is a fundamental spherical distribution for axial data. The probability density function of a Watson distribution on *S* ^2^ is [27]

$$p\left(\pm n_{i};\mu ,\kappa \right)=M{\left(\frac{1}{2},\frac{3}{2},\kappa \right)}^{-1}e^{\kappa {\left(\mu^{T}n_{i}\right)}^{2}}.$$

Here, $M{\left(\frac{1}{2},\frac{3}{2},\cdot \right)}^{-1}$ is the Kummer function [27], ***μ***(||***μ***|| = 1) is the population mean axis, and *κ* is a concentration parameter. For *κ* > 0, the distribution is bipolar and *κ* characterizes how strongly the unit vectors are concentrated around the mean orientation (larger *κ* means more concentrated around ±***μ***).

## Supporting Information

S1 FigInfluence of $T_{c_{l}}$ on a set of three extracted bundles.The CC, the Cg, and the CST. In (a), the opacity axis is left-right oriented, which renders pathways with a global left-right direction (e.g. the lateral (fanning) projections of the CC) transparent. When applying a *c*_*l*_ threshold, streamlines that both have a left-right orientation and a high dispersion (i.e., low *c*_*l*_) are rendered opaque. When increasing $T_{c_{l}}$ (towards the right), streamlines with an increasingly high *c*_*l*_ are rendered opaque. In (b), the opacity axis is antero-posterior oriented. Increasing $T_{c_{l}}$ results in the display of a larger amount of curved Cg streamlines. Opacity function *α*_decreasing_ with *c* = 3 was used in all figures.(PNG)

S2 Fig Local transparency rendering with a piecewise linear function. *α*(*θ*) = *aθ*+*b* if *θ*≥-*b*/*a* ∧ *θ*≤(1−*b*)/*a* *α*(*θ*) = 1 if *θ*>(1−*b*)/*a*, and *α*(*θ*) = 0 if *θ*<-*b*/*a*, with *θ* = acos|*n*·*t*|ϵ[0,*π*/2], the opacity increases when the angle increases. Graphs of the opacity function and the corresponding renderings are shown for different values of *a* and *b*. Parameter *b* appears to have the largest influence on the visualization, whereas *a* only smoothens the transitions between transparent and opaque streamline segments (see subtle differences highlighted by the white squares).
(PNG)

S3 Fig Histograms of |*n*·*t*| as a function of the opacity axis. (left-right (left), antero-posterior (middle), inferior-superior (right) direction). (a) Locally and (b) Globally defined orientations.
(PNG)
