# Supplementary figures and images for: Seeing More by Showing Less: Orientation-Dependent Transparency Rendering for Fiber Tractography Visualization
Source: PLoS One. 2015 Oct 7;10(10):e0139434. doi: 10.1371/journal.pone.0139434 (PMC4596805; doi:10.1371/journal.pone.0139434)

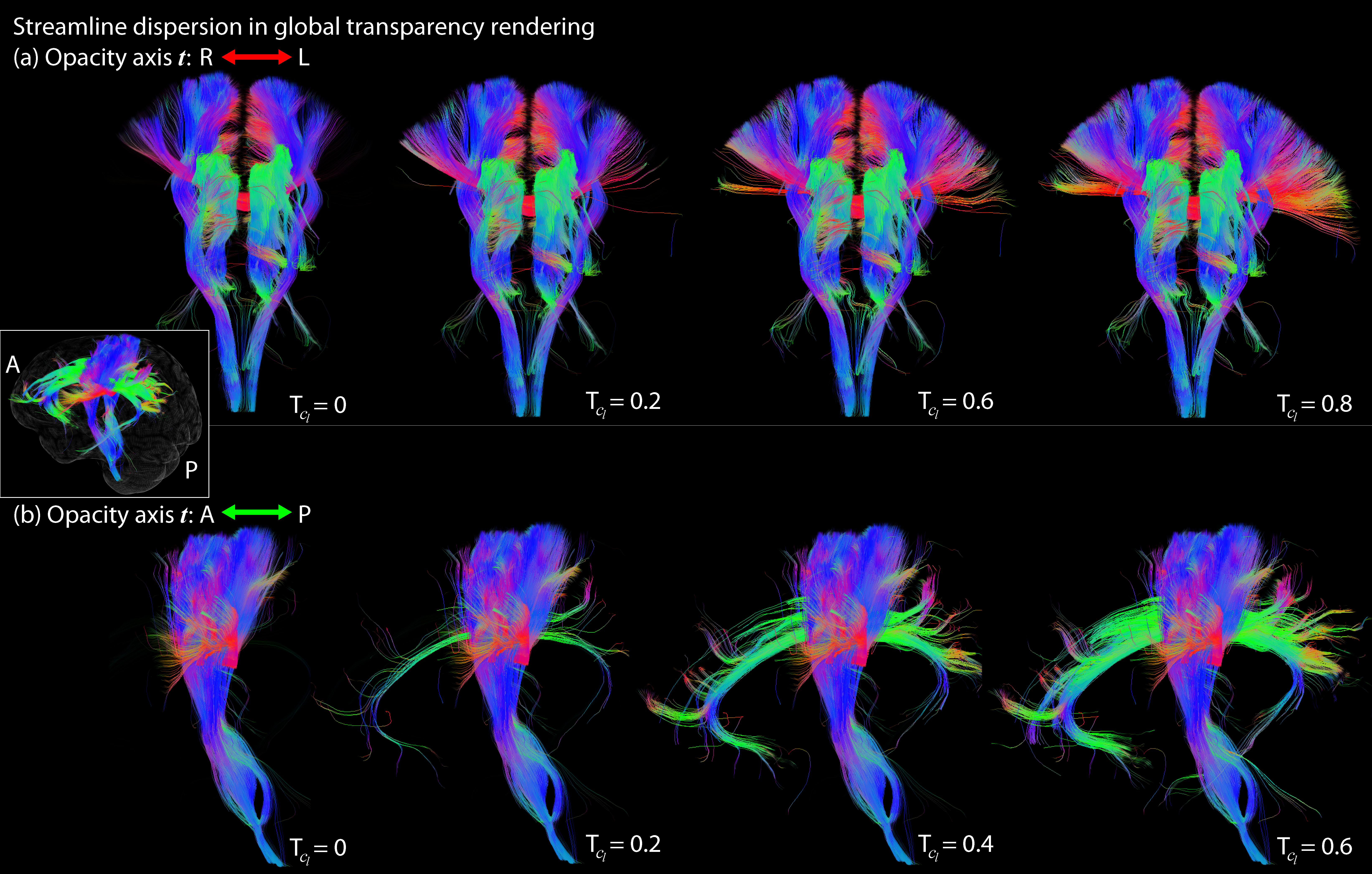

Supplement: S1 Fig — The CC, the Cg, and the CST. In (a), the opacity axis is left-right oriented, which renders pathways with a global left-right direction (e.g. the lateral (fanning) projections of the CC) transparent. When applying a c l threshold, streamlines that both have a left-right orientation and a high dispersion (i.e., low c l) are rendered opaque. When increasing Tcl (towards the right), streamlines with an increasingly high c l are rendered opaque. In (b), the opacity axis is antero-posterior oriented. Increasing Tcl results in the display of a larger amount of curved Cg streamlines. Opacity function α decreasing with c = 3 was used in all figures. (PNG) [file pone.0139434.s001.png]

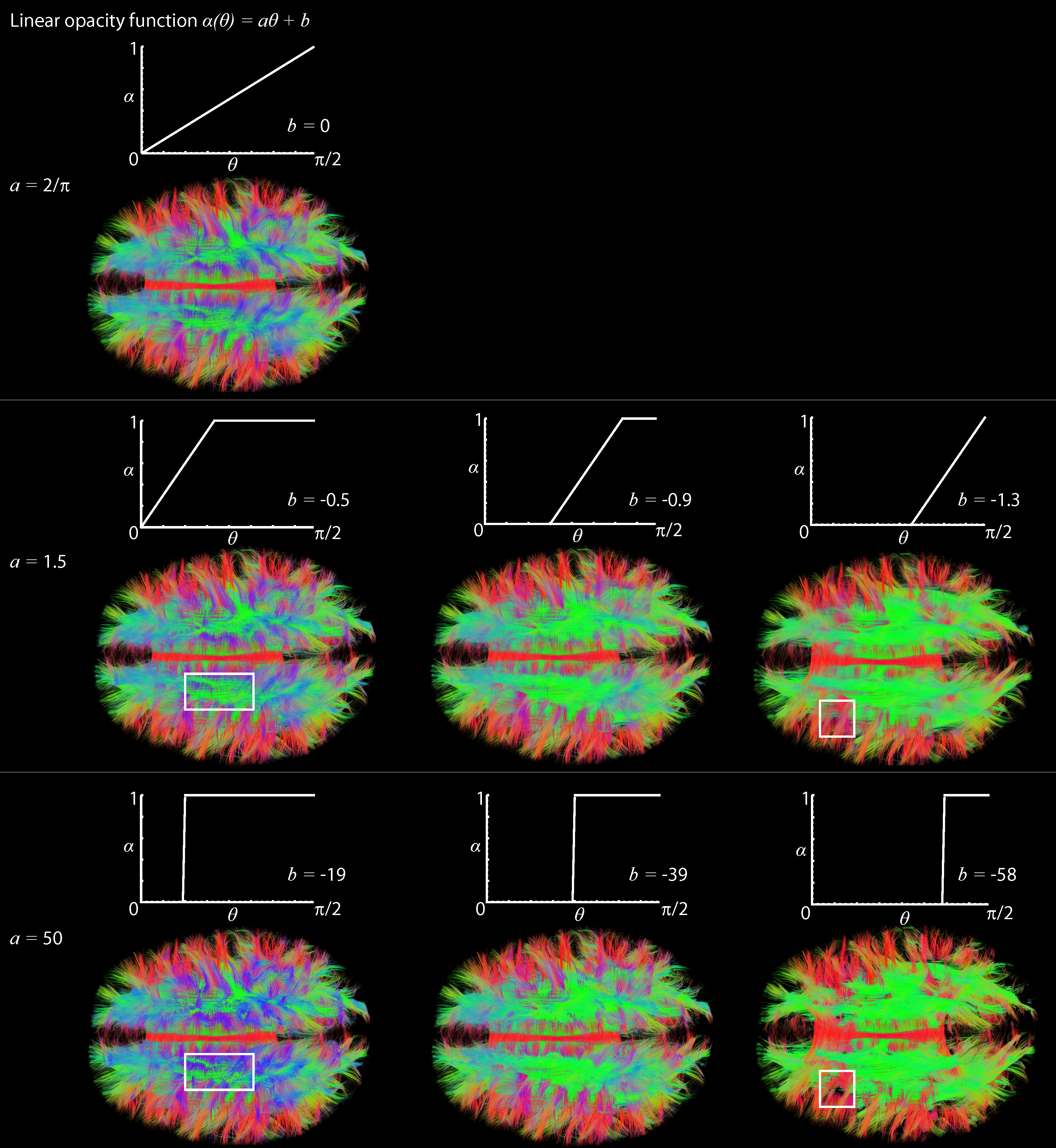

Supplement: S2 Fig — α(θ) = aθ+b if θ≥-b/a ∧ θ≤(1−b)/a α(θ) = 1 if θ>(1−b)/a, and α(θ) = 0 if θ<-b/a, with θ = acos|n·t|ϵ[0,π/2], the opacity increases when the angle increases. Graphs of the opacity function and the corresponding renderings are shown for different values of a and b. Parameter b appears to have the largest influence on the visualization, whereas a only smoothens the transitions between transparent and opaque streamline segments (see subtle differences highlighted by the white squares). (PNG) [file pone.0139434.s002.png]

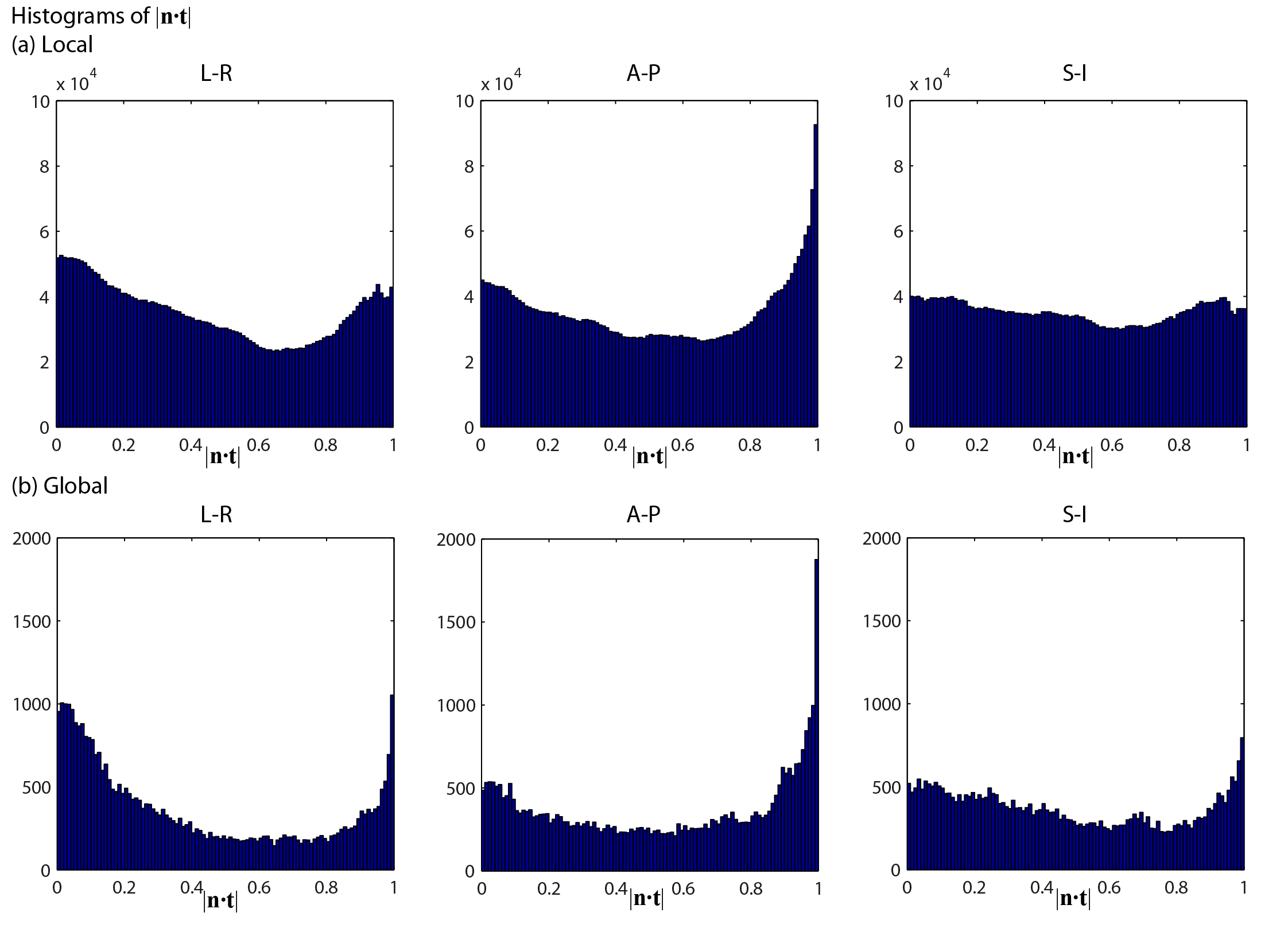

Supplement: S3 Fig — (left-right (left), antero-posterior (middle), inferior-superior (right) direction). (a) Locally and (b) Globally defined orientations. (PNG) [file pone.0139434.s003.png]
